# Assessing post-COVID-19 respiratory dynamics: a comprehensive analysis of pulmonary function, bronchial hyperresponsiveness and bronchodilator response

**DOI:** 10.1183/23120541.00149-2024

**Published:** 2024-10-07

**Authors:** Chun-Yao Huang, Yao-Kuang Wu, Mei-Chen Yang, Kuo-Liang Huang, Wen-Lin Su, Yi-Chih Huang, Wu Chih-Wei, I-Shiang Tzeng, Chou-Chin Lan

**Affiliations:** 1 Division of Pulmonary Medicine, Taipei Tzu Chi Hospital, Buddhist Tzu Chi Medical Foundation, New Taipei City, Taiwan; 2 School of Medicine, Tzu Chi University, Hualien, Taiwan; 3 Department of Research, Taipei Tzu Chi Hospital, Buddhist Tzu Chi Medical Foundation, New Taipei City, Taiwan

## Abstract

**Background:**

Coronavirus disease 2019 (COVID-19) has a considerable impact on the global healthcare system. Individuals who have recovered from COVID often experience chronic respiratory symptoms that affect their daily lives. This study aimed to assess respiratory dynamics such as airway hyperresponsiveness (AHR) and bronchodilator response in post-COVID patients.

**Methods:**

This study included 282 adults with respiratory symptoms who underwent provocation tests. The demographic details, clinical symptoms and medical histories were recorded. Baseline spirometry, methacholine challenge tests (MCT) and post-bronchodilator spirometry were performed. Patients were divided into the following four groups: Group 1: non-COVID-19 and negative MCT; Group 2: post-COVID-19 and negative MCT; Group 3: non-COVID-19 and positive MCT; and Group 4: post-COVID-19 and positive MCT.

**Results:**

Most post-COVID-19 patients (43.7%) experienced AHR, and wheezing was more common. Patients in Group 4 exhibited increased intensities of dyspnoea, cough and wheezing with the lowest pulmonary function test (PFT) parameters at baseline. Moreover, significant decreases in PFT parameters after the MCT were observed in these patients. Although the prevalence of a low forced expiratory volume in 1 s to forced vital capacity ratio (<70%) was initially 2% in Group 4, it increased to 29% after MCT. No significant differences in allergic history or underlying diseases were observed between the groups.

**Conclusions:**

These findings provide comprehensive insights into the AHR and respiratory symptoms of post-COVID-19 individuals, highlighting the characteristics and potential exacerbations in patients with positive MCT results. This emphasises the need of MCT to address respiratory dynamics in post-COVID-19 individuals.

## Shareable abstract

This study reveals post-COVID-19 patients often face AHR, with worsened respiratory symptoms like cough and dyspnoea. Methacholine tests are essential for diagnosis and tailoring treatments, emphasising the need for targeted post-COVID-19 care. https://bit.ly/4bBpWKq

## Introduction

Coronavirus disease-19 (COVID-19) has a profound impact on the global health system, overwhelming medical facilities and resources [1, 2]. Despite the considerable impact of COVID-19, the health system continues to undergo substantial transformations in the post-pandemic era. Excessive mortality rates have been observed in the aftermath of the COVID-19 pandemic [3]. Therefore, persistent attention to the long-term health outcomes of individuals who have experienced post-COVID conditions remains vital.

Many survivors of COVID-19 exhibited chronic symptoms, with ∼43% reporting persistent respiratory symptoms [4]. Dyspnoea is an independent predictor of morbidity and mortality and is related to reduced functional capacity and compromised health-related quality of life [5, 6]. Persistent coughing can evolve into a troublesome issue that may spiral into a vicious cycle, as this could exacerbate irritation, further intensifying the cough [7]. These complex symptoms may lead to a cycle of deconditioning, and difficulty performing daily activities, emphasising the need for further research to unravel the mechanisms underlying persistent respiratory symptoms in post-COVID-19 survivors [5].

Airway hyperresponsiveness (AHR) should be considered a potential factor in patients with persistent cough or dyspnoea [8]. Employing objective tests can prove beneficial for achieving accurate diagnoses. Methacholine challenge testing (MCT) is highly sensitive and has a substantial negative predictive value for diagnosing and assessing AHR [9, 10]. These tests induce specific responses, providing objective measurements that are crucial for tailoring treatment strategy [10].

Numerous post-COVID-19 patients experience chronic respiratory symptoms that affect their daily activities and quality of life. However, the extent of the AHR in these patients remains unclear. This study aimed to explore the post-COVID-19 respiratory dynamics involved analysing the respiratory symptoms, lung function parameters, AHR and bronchodilator response to provide valuable insights into the post-COVID-19 respiratory landscape.

## Materials and methods

### Study design and patient selection

We retrospectively analysed patients who underwent provocation tests at an outpatient clinical department specialising in pulmonary medicine between 1 May 2022 and 30 September 2022. All these patients were referred from the pulmonology outpatient clinic. Our study enrolled patients aged 18 years and older who exhibited chronic respiratory symptoms, including cough, wheeze or dyspnoea, and expressed a willingness to participate. We excluded individuals younger than 18 years, those without respiratory symptoms and those unwilling to be included in the study. Prior to participating in this study, all patients were only treated with symptomatic relief medications, such as cough suppressants, expectorants and antihistamines. No one had used steroids or bronchodilators.

The study cohort comprised 282 adults who experienced respiratory symptoms. All patients underwent an assessment that included demographic details such as age, sex and body mass index (BMI). The study protocol was approved by the Ethics Committee of Taipei Tzu Chi Hospital. Informed consent was obtained from all the patients.

### Clinical variables

Clinical variables included demographics such as BMI, smoking status and allergic history (including allergies to food or drugs). Additionally, information on the family history of asthma was documented. Medical histories of the underlying conditions, such as tuberculosis, COPD, bronchiectasis, idiopathic pulmonary fibrosis, congestive heart failure (CHF), coronary artery disease (CAD), renal disease, liver disease, hypertension, diabetes mellitus (DM) and cancer, were recorded.

### Methacholine challenge test

#### Baseline spirometry

Pulmonary function tests (PFTs) were measured using a spirometer (Medical Graphics Corp., St. Paul, MN, USA) in accordance with the American Thoracic Society guidelines [11]. To ensure the non-COVID group remained unaffected by COVID-19 during the MCT, PFTs were performed in a negative pressure room, reducing airborne transmission risks from SARS-CoV-2 and various viruses [9]. The environment was thoroughly disinfected after each session to ensure safety for subsequent patients and staff. Furthermore, prior to testing, patients underwent screening for symptoms indicative of acute infections, such as fever. Those exhibiting any signs of acute infection were excluded from participating in the PFTs [9].

The reference equations for calculating the predicted values of spirometric parameters were based on a prior study in Taiwan [12]. For males, the equations were as follows: forced vital capacity (FVC, in litres) = −3.09 − 0.030 * age + 0.050 * body height (BH, in cm); forced expiratory volume in 1 s (FEV_1_, in litres) = −1.646 − 0.031 * age + 0.038 * BH; and maximum mid-expiratory flow (MMEF, in litres per second) = 1.047 − 0.047 * age + 0.029 * BH. For females, the equations were: FVC= −2.170 − 0.019 * age + 0.038 * BH; FEV_1_ = −1.123 − 0.021 * age + 0.029 * BH; and MMEF = 1.464 − 0.034 * age + 0.020 * BH [12]. The FEV_1_/FVC ratio is the measured value of FEV_1_ divided by the measured value of FVC. A low FEV_1_/FVC ratio is defined as an FEV_1_/FVC ratio (%) <70% [13].

#### Post-MCT spirometry

MCT was performed using a methacholine solution in phosphate-buffered saline [9]. PFT measurements were performed at baseline and post-inhalation methacholine with concentrations ranging from 0.0625 mg·mL^−1^ to 16.0 mg·mL^−1^ [9]. The methacholine was administered using a 5-breath deep breathing technique, with the nose clipped *via* an AeroEclipse II Breath-actuated Nebulizer from (Trudell Medicinal International, Ontario, Canada) [14]. Post-methacholine PFT was performed after 3 min of methacholine administration. The changes in FVC (ΔFVC), FEV_1_ (ΔFEV_1_) and MMEF (ΔMMEF) after methacholine challenge were calculated as the difference between post-methacholine and baseline values divided by the baseline values. Provocative concentration (PC20) refers to the concentration of methacholine that causes a 20% reduction in FEV_1_ [15].

#### Post-bronchodilator spirometry

Following the methacholine challenge, a bronchodilator, fenoterol (Berotec 100, Boehringer Ingelheim), at a dose of 200 mg, was delivered *via* a pressurised metered-dose inhaler. Post-bronchodilator spirometry was performed 15 min after fenoterol administration. The changes in FVC (ΔFVC), FEV_1_ (ΔFEV_1_) and MMEF (ΔMMEF) after fenoterol administration were calculated as the difference between the post-bronchodilator and post-MCT values, divided by the post-MCT values.

### Symptoms severity score

Physicians reviewed the respiratory symptoms while patients reported insights on the severity at an outpatient clinical department. The severity for all reported symptoms was categorised into five categories: none (score 0), mild (score 1), moderate (score 2), severe (score 3) or very severe (score 4).

### Statistical analysis

All statistical analyses were performed using SPSS 24.0 software (SPSS Inc., Chicago, IL, USA). Chi-square tests were applied to analyse the categorical variables of the four groups or specific two groups. The Shapiro–Wilk test was employed to assess the normality of continuous variables. Variables conforming to normal distribution were then analysed using analysis of variance to evaluate the significance of mean differences for the four groups, and *post hoc* comparisons were performed using the least significant difference method to identify specific group differences. These data are presented as mean±sd. For variables that did not exhibit normality, the Kruskal–Wallis test was utilised to evaluate the significance of differences in medians, and findings are reported as median and interquartile range. The statistical significance was set at p<0.05.

## Results

This study analysed 282 patients who were divided into four groups: Group 1: non-COVID-19 and negative MCT (n=108); Group 2: post-COVID-19 and negative MCT (n=49); Group 3: non-COVID-19 and positive MCT (n=87); and Group 4: post-COVID-19 and positive MCT (n=38). Among non-COVID patients, 44.6% tested positive for MCT, which was similar to that in post-COVID patients (43.7% MCT positivity rate; p>0.05).

### Baseline demographic characteristics, underlying disease and allergic history

Table 1 summarises the baseline characteristics. Age, BMI, sex distribution and smoking status were comparable between the groups (p>0.05 for all). Time since COVID-19 infection was 58.5±36.0 days in Group 2 and 61.6±38.4 days in Group 2. COVID-19 vaccination rates were 4.6% in Group 1, 6.1% in Group 2, 13.8% in Group 3 and 0% in Group 4, with Group 3 having the highest vaccination rate (p=0.009). All the patients had mild cases of COVID-19 and were not hospitalised, did not receive oxygen therapy, or were not put on a noninvasive ventilator or mechanical ventilator.

**TABLE 1:** Baseline characteristics and underlying diseases of post-COVID-19 and non-COVID-19 patients with positive or negative provocation test results

	Group 1: COVID (−), MCT (−)	Group 2: COVID (+), MCT (−)	Group 3: COVID (−), MCT (+)	Group 4: COVID (+), MCT (+)	p-value
**Patients n**	108	49	87	38	
**Age years**	57.0 (38.8–66.3)	47.0 (40.0–59.0)	53.0 (40.5–65.0)	47.0 (33.8–59.8)	0.158
**BMI kg·m^−2^**	23.6 (21.2–26.1)	23.4 (21.2–26.5)	23.3 (21.2–26.6)	22.0 (20.4–25.2)	0.441
**Male/female, n/N (%)**	42/66 (39/61)	15/34 (31/69)	25/82 (29/71)	10/28 (26/74)	0.347
**Time after COVID-19 infection days**	0±0^#,¶^	58.5±36.0^#,+^	0±0^+,§^	61.6±38.4^¶,§^	<0.001
**COVID-19 Vaccination, n (%)**	5 (4.6)^ƒ^	3 (6.1)	12 (13.8)^ƒ,§^	0 (0)^§^	0.009
**Current smoker/never-smoker, n/N (%)**	6/102 (6/94)	2/47 (4/96)	13/74 (15/85)	3/35 (8/92)	0.210
**Tobacco consumption pack-years**	1.1±5.9	1.6±6.6	2.3±11.3	1.7±8.1	0.834
**Food allergy, n (%)**	5 (5)	2 (4)	6 (7)	3 (8)	0.790
**Drug allergy, n (%)**	14 (13)	11 (22)	19 (22)	2 (5)	0.055
**FHx of asthma, n (%)**	28 (26)	11 (22)	26 (30)	6 (16)	0.387
**TB, n (%)**	3 (3)	1 (2)	0 (0)	0 (0)	0.339
**COPD, n (%)**	16 (15)	7 (14)	7 (8)	0 (0)	0.050
**Bronchiectasis, n (%)**	5 (5)	0 (0)	2 (2)	0 (0)	0.232
**IPF, n (%)**	1 (1)	0 (0)	1 (1)	0 (0)	0.817
**CHF, n (%)**	6 (6)	0 (0)	3 (3)	2 (5)	0.390
**CAD, n (%)**	6 (6)	2 (4)	4 (5)	3 (8)	0.861
**Renal disease, n (%)**	1 (1)	0 (0)	0 (0)	1 (5)	0.059
**Liver disease, n (%)**	4 (4)	2 (4)	4 (5)	1 (3)	0.962
**Hypertension, n (%)**	23 (21)	6 (12)	18 (21)	6 (16)	0.522
**DM, n (%)**	10 (9)	3 (6)	7 (8)	5 (13)	0.702
**Cancer, n (%)**	4 (4)	0 (0)	4 (5)	0 (0)	0.282

Note: Continuous data are presented as mean±sd or median with first and third quantiles according to their normality. MCT: methacholine challenge test; BMI: body mass index; FHx: family history; TB: tuberculosis; IPF: idiopathic pulmonary fibrosis; CHF: congestive heart failure; CAD: coronary artery disease; DM: diabetes mellitus. ^#^: p<0.05 as comparison between COVID (−), MCT (−) and COVID (+), MCT (−); ^¶^: p<0.05 as comparison between COVID (−), MCT (−) and COVID (+), MCT (+); ^+^: p<0.05 as comparison between COVID (+), MCT (−) and COVID (−), MCT (+); ^§^: p<0.05 as comparison between COVID (−), MCT (−) and COVID (+), MCT (+); ^ƒ^: p<0.05 as comparison between COVID (−), MCT (−) and COVID (−), MCT (+).

The prevalence of food allergy ranged between 4% and 8%, with no significant differences between the groups (p=0.790). Variations in drug allergy were observed between the groups but without significant differences (Groups 1, 2, 3 and 4 at 13%, 22%, 22% and 5%, respectively) (p=0.055). Family history of asthma ranged from 16% to 30%, without significant differences (p=0.387). Overall, allergic histories did not significantly differ among the groups. COPD showed distinctions in comorbidities, with Groups 1, 2, 3 and 4 at 15%, 14%, 8% and 0%, respectively, approaching significance (p=0.050). Bronchiectasis, CHF, CAD, renal disease, liver disease, hypertension, DM and cancer did not differ significantly between groups (all p>0.05). No patient has previously documented asthma.

### Clinical symptoms

Table 2 presents a comparison of symptoms between patients. The prevalence of wheezing differed significantly between the groups, with the highest prevalence in Group 4 (45%, p<0.001). No significant differences were observed in the prevalence of cough (64–65%), chest tightness (46–61%) or dyspnoea (34–49%) between the groups. Severity assessments of cough and sputum revealed no significant differences between the groups. However, dyspnoea, chest tightness and wheezing were higher in Group 4 (dyspnoea: 1.0±1.0, chest tightness: 1.1±1.1, wheezing: 0.4±1.0) than in other groups (all p<0.05 by *post hoc* analysis).

**TABLE 2:** Symptoms of post-COVID-19 and non-COVID-19 patients with positive or negative provocation test results

	Group 1: COVID (−), MCT (−)	Group 2: COVID (+), MCT (−)	Group 3: COVID (−), MCT (+)	Group 4: COVID (+), MCT (+)	p-value
**Subjects n**	108	49	87	38	
**Prevalence of symptoms, n (%)**					
** **Wheezing	14 (13)^#,¶^	8 (16)^+,§^	31 (36)^#,+^	17 (45)^¶,§^	<0.001
** **Cough	69 (64)	32 (65)	55 (63)	24 (63)	0.995
** **Chest tightness	50 (46)	24 (49)	43 (49)	23 (61)	0.514
** **Dyspnoea	39 (36)	18 (37)	30 (49)	30 (34)	0.961
**Severity of symptoms, mean±sd**					
** **Cough	1.5±1.1	1.4±1.0	1.6±0.9	1.4±0.9	0.449
** **Sputum	1.2±1.1	1.0±1.0	1.2±1.1	1.0±1.0	0.732
** **Dyspnoea	0.5±0.8^¶^	0.6±0.7	0.6±0.8	1.0±1.0^¶^	0.096
** **Chest tightness	0.7±0.9^¶^	0.7±0.8^§^	0.9±0.9	1.1±1.1^¶,§^	0.069
** **Wheezing	0.2±0.6	0.1±0.4^§^	0.1±0.5^ƒ^	0.4±1.0^§,ƒ^	0.079

MCT: methacholine challenge test. ^#^: p<0.05 as comparison between COVID (−), MCT (−) and COVID (−), MCT (+); ^¶^: p<0.05 as comparison between COVID (−), MCT (−) and COVID (+), MCT (+); ^+^: p<0.05 as comparison between COVID (+), MCT (−) and COVID (−), MCT (+); ^§^: p<0.05 as comparison between COVID (−), MCT (+) and COVID (+), MCT (+); ^ƒ^: p: <0.05 as comparison between COVID (+), MCT (−) and COVID (+), MCT (+).

### PFT at baseline, post-MCT and post-bronchodilator

Table 3 shows the PFT results at baseline, post-MCT and post-bronchodilator administration. At baseline, FVC was lower in Groups 3 and 4 (2.9±0.9 L, 88.3±14.7%; 2.9±0.9 L, 83.4±12.3%) than in Groups 1 and 2 (3.2±1.0 L, 94.0±14.1%; 3.2±0.9 L, 93.2±12.9%) (p<0.05). Similar differences were observed in FEV_1_ and MMEF (%) (both p<0.001), with Group 4 consistently demonstrating the lowest values.

**TABLE 3:** Provocation test between post-COVID-19 and non-COVID-19 with positive or negative provocation test results

	Group 1: COVID (−), MCT (−)	Group 2: COVID (+), MCT (−)	Group 3: COVID (−), MCT (+)	Group 4: COVID (+), MCT (+)	p-value
**Patients n**	108	49	87	38	
**Baseline**					
** **FVC L	3.2±1.0^#,¶^	3.2±0.9^+,§^	2.9±0.9^#,+^	2.9±0.9^¶,§^	0.002
** **FVC % predicted	94.0±14.1^#,¶^	93.2±12.9^+,§^	88.3±14.7^#,+^	83.4±12.3^¶,§^	<0.001
** **FEV_1_ L	2.6±0.8^#,¶^	2.7±0.8^+,§^	2.3±0.8^#,+^	2.3±0.8^¶,§^	<0.001
** **FEV_1_ % predicted	94.6±13.9^#,¶^	95.3±12.5^+,§^	85.2±16.9^#,+^	82.2±14.8^¶,§^	<0.001
** **FEV_1_/FVC %	82.7±5.9^#^	84.7±5.1^+,§^	79.1±8.7^#,+^	81.1±8.7^§^	<0.001
** **MMEF L·s^−1^	2.8±1.1^#,¶^	3.1±1.3^+,§^	2.2±1.2^#,+^	2.3±1.1^¶,§^	<0.001
** **MMEF % predicted	91.3±22.6^#,¶^	97.4±23.6^+,§^	74.9±29.0^#,+^	73.8±25.0^¶,§^	<0.001
**Post-MCT**					
** **FVC L	3.0±0.9^#,¶^	3.1±0.9^+,§^	2.4±0.8^#,+^	2.3±0.8^¶,§^	0.019
** **FVC % predicted	88.2±14.4^#,¶^	87.7±14.0^+,§^	73.3±13.5^#,+^	68.1±13.5^¶,§^	<0.001
** **FEV_1_ L	2.4±0.7^#,¶^	2.5±0.8^+,§^	1.7±0.6^#,+^	1.7±0.6^¶,§^	0.001
** **FEV_1_ % predicted	85.1±14.1^#,¶^	86.1±13.7^+,§^	64.6±13.6^#,+,ƒ^	58.4±13.0^¶,§,ƒ^	<0.001
** **FEV_1_/FVC %	79.2±5.7^#,¶^	81.3±6.1^+,§^	73.6±8.0^#,+^	71.2 ±8.6^¶,§^	<0.001
** **MMEF L·s^−1^	1.8±0.9^#,¶^	2.2±1.4^+,§^	0.8±0.8^#,+^	0.8±0.5^¶,§^	<0.001
** **MMEF % predicted	60.0±21.8^#,¶^	67.2±30.0^+,§^	25.3±14.2^#,+^	24.4±13.1^¶,§^	<0.001
** **ΔFVC %	−6.2±4.6^#,¶^	−6.0±5.9^+,§^	−16.9±6.8^#,+^	−18.8±8.4^¶,§^	<0.001
** **ΔFEV_1_ %	−10.2±5.2^#,¶^	−9.8±5.3^+,§^	−23.8±5.8^#,+,ƒ^	−28.6±8.6^¶,§,ƒ^	<0.001
** **ΔMMEF %	−34.8±16.9^#,¶^	−33.5±17.8^+,§^	−62.1±37.6^#,+^	−66.8±11.0^¶,§^	<0.001
**Post-bronchodilator spirometry**					
** **FVC L	3.1±1.0^#,¶^	3.2±0.9^+,§^	2.8±0.9^#,+^	2.7±0.9^¶,§^	0.001
** **FVC % predicted	91.9±14.4^#,¶^	90.8±13.3^+,§^	85.7±14.1^#,+^	80.0±12.7^¶,§^	<0.001
** **FEV_1_ L	2.6±0.8^#,¶^	2.7±0.8^+,§^	2.2±0.7^#,+^	2.2±0.8^¶,§^	<0.001
** **FEV_1_ % predicted	91.9±14.3^#^	92.1±12.2^+,§^	82.0±15.0^#,+^	77.5±14.0^¶,§^	0.001
** **FEV_1_/FVC %	82.5±5.7^#,¶^	84.1±5.0	78.0±10.1^#^	80.4±8.8	<0.001
** **MMEF L·s^−1^	2.5±1.0^#,¶^	2.7±1.3^+,§^	2.0±1.1^#,+^	2.0±1.1^¶,§^	<0.001
** **MMEF % predicted	82.8±23.9^#,¶^	85.2±24.7^+,§^	65.3±26.8^#,+^	60.1±23.6^¶,§^	<0.001
** **ΔFVC (%)	4.4±4.2^#,¶^	3.8±4.8^+,§^	17.8±10.7^#,+^	18.7±13.6^¶,§^	<0.001
** **ΔFEV_1_ (%)	8.4±5.4^#,¶^	7.4±5.7^+,§^	26.8±16.0^#,+^	35.0±22.8^¶,§^	<0.001
** **ΔMMEF (%)	47.0±39.7^#,¶^	38.4±38.8^+,§^	191.0±125.8^#,+^	167.8±85.6^¶,§^	<0.001

Data are presented as mean±sd. MCT: methacholine challenge test; FVC: forced vital capacity; FEV_1_: forced expiratory volume in 1 s; MMEF: maximum mid-expiratory flow. ^#^: p<0.05 as comparison between COVID (−), MCT (−) and COVID (−), MCT (+); ^¶^: p<0.05 as comparison between COVID (−), MCT (−) and COVID (+), MCT (+); ^+^: p<0.05 as comparison between COVID (+), MCT (−) and COVID (−), MCT (+); ^§^: p<0.05 as comparison between COVID (+), MCT (−) and COVID (+), MCT (+); ^ƒ^: p<0.05 as comparison between COVID (−), MCT (+) and COVID (+), MCT (+).

Post-MCT, FVC remained lower in Group 3 (2.4±0.8 L, 73.3±13.5%) and 4 (2.3±0.8 L, 68.1±13.5%) than in Group 1 and 2 (3.0±0.9 L, 88.2±14.4%; 3.1±0.9 L, 87.7±14.0%) (p<0.05). Changes in FVC (%), FEV_1_ (%), and MMEF (%) post-MCT were most pronounced in Group 4, indicating increased respiratory dysfunction in post-COVID-19 patients with positive MCT results. For patients in Groups 3 and 4, the PC20 showed a trend towards increased AHR (4.8±3.4 mg·mL^−1^ and 3.5±3.0 mg·mL^−1^ in Group 3 and Group 4, respectively) (p=0.082).

Post-bronchodilator FVC remained lower in Group 3 (2.8±0.9 L, 85.7±14.1%) and 4 (2.7±0.9 L, 80.0±12.7%) than in Groups 1 and 2 (3.1±1.0 L, 91.9±14.4%; 3.2±0.9 L, 90.8±13.3%) (p<0.05). Groups 3 and 4 showed significant improvements in FVC, FEV_1_ and MMEF, indicating a pronounced bronchodilator response in post-COVID-19 patients with positive MCT results.

### Prevalence of low FEV_1_/FVC ratio

**FIGURE 1:**
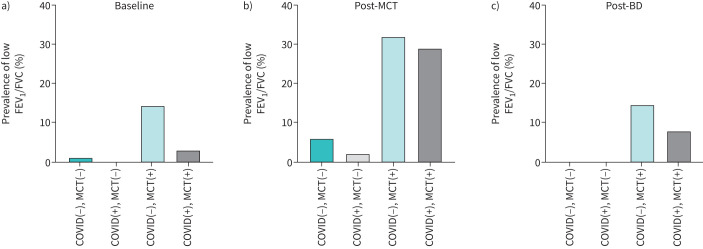
Prevalence of low forced expiratory volume in 1 s (FEV_1_)/forced vital capacity (FVC) between post-COVID-19 and non-COVID-19 with positive or negative provocation test results. a) Baseline; b) post-methacholine challenge test (MCT); and c) post-bronchodilator (BD).

Figure 1 shows the prevalence of a low FEV_1_/FVC ratio (<70%). Non-COVID patients with a negative MCT in the cohort had a baseline prevalence of 1% (figure 1a), whereas the prevalence was 0% in post-COVID-19 patients with a negative MCT. The baseline prevalence of low FEV_1_/FVC was the highest in non-COVID-19 patients with positive MCT (14%), compared to 3% in post-COVID-19 patients with positive MCT. Following MCT (figure 1b), the prevalence of low FEV_1_/FVC increased in all groups; 32% and 29% increase in patients with non-COVID-19 positive MCT and COVID-19 positive MCT, respectively. After bronchodilator administration (figure 1c), the prevalence decreased in those without COVID-19 and negative MCT (to 0%), with similar reductions in the other groups (0–8%).

## Discussion

There are many important findings in the current study. Among patients of post-COVID-19 with chronic respiratory symptoms, 43.7% of patients tested positive for MCT. Wheezing was the most common clinical symptom, and the severity of dyspnoea, cough and wheezing was more in patients with post-COVID-19 with AHR. Baseline PFT revealed that post-COVID-19 patients with AHR exhibited the lowest PFT values, suggesting compromised respiratory function in this subgroup. Post-COVID-19 patients with AHR also showed significant decreases in pulmonary function after MCT, indicating more airway hypersensitivity. The prevalence of a low FEV_1_/FVC ratio increased after the MCT in patients with post-COVID-19 with AHR. Overall, these findings provide comprehensive insights into the respiratory symptoms and AHR of patients with post-COVID-19, highlighting the specific characteristics and potential exacerbations in patients.

In a recent study, among 394 post-COVID-19 patients with respiratory symptoms, 36 were suspected to have asthma [16]. Within this group, new-onset asthma diagnoses were made for 17 individuals. Therefore, it was reported that 4.3% of patients with post-COVID-19 infection were diagnosed with new-onset asthma [16]. However, since not all patients underwent a MCT [16], the prevalence of diagnosed asthma may be underestimated. In our study, ∼40% of the post-COVID patients with chronic respiratory symptoms had asthma. An abstract on AHR in COVID-19 presented at the 2022 ERS International Congress reported that 43% of COVID-19 patients tested positive for MCT, which is lower than in those without COVID-19, 56% of whom exhibited MCT positivity [17]. Dyspnoea is a common complaint often associated with AHR in patients with a history of COVID-19 [17]. Our findings indicate that patients with post-COVID-19 and AHR experienced symptoms other than dyspnoea, such as increased chest tightness and wheezing. Furthermore, post-COVID patients with AHR had lower PFT scores. Post-COVID patients with AHR exhibited a lower PC20 in comparison to non-COVID patients with AHR, although the difference was not statistically significant. Therefore, patients with post-COVID-19 and AHR manifested increased airway hypersensitivity and respiratory symptoms. Although the mechanism of association between COVID-19 and AHR remains unclear, some studies have implicated eosinophilic inflammation as a potential causative factor [18].

Various treatments have been used to address persistent COVID-related respiratory symptoms [7]. Empirical administration of inhaled steroids are suggested for patients experiencing persistent cough after COVID, although their efficacy remains unknown [7]. However, AHR was not observed in all the post-COVID-19 patients with chronic respiratory symptoms. Therefore, we recommend considering MCT before initiating the administration of inhaled steroids. When persistent respiratory symptoms commonly observed in post-COVID-19 patients do not respond to symptom-relieving treatments, clinicians should consider asthma as a potential diagnosis [16].

Therefore, it is important to determine whether patients who have recovered from COVID-19 should undergo PFT. In a previous study, patients with mild COVID-19 infections generally did not show abnormalities in PFT, suggesting that follow-up PFT may not be necessary [19]. In another study, impulse oscillometry was used to assess small airway resistance in 38 patients with post-COVID-19 experiencing post-infection cough compared with a control group [20]. The results revealed no statistically significant differences in respiratory parameters between the groups. They suggested that the small airways were not adversely affected by COVID-19 [20]. In the current study, of the post-COVID patients with positive MCT, 97% exhibited normal FEV_1_/FVC. However, after MCT, the number of patients with lower FEV_1_/FVC increased to 29%. These results suggest that AHR remains an important concern in post-COVID-19 patients. Notably, simple spirometry or impulse oscillometry without challenges may not be sufficiently accurate as diagnostic tools to evaluate airway conditions in post-COVID-19 patients.

In the current study, patients with chronic respiratory symptoms and positive MCT exhibited reduced baseline FEV_1_ and FVC regardless of their COVID-19 status. These findings underscore the impaired pulmonary function in patients with AHR. The reduction in FEV_1_ and FVC suggested a constricted airway [21]. This highlights the importance of spirometric evaluation and MCT in the diagnostic workup for individuals with chronic respiratory complaints regardless of COVID-19 infection or not. The MCT may provide further insights into the extent of respiratory impairment [21].

Patients with chronic respiratory symptoms and positive MCT results present similarly, regardless of COVID-19 status. Previous studies have indicated that COVID-19 infection can cause airway inflammation and augmented AHR [22]. Other viral infections and gene–environment interactions may also contribute to these conditions. Various risk factors, including microbial exposure, pollution, smoking and family history, play critical roles in the pathogenesis of asthma [23]. Non-COVID viral infections such as Epstein–Barr virus, cytomegalovirus and influenza virus are also known to induce airway inflammation and AHR [24]. Therefore, it is rational that both COVID-19 and non-COVID factors are implicated in airway inflammation. Persistent inflammation and hyperreactivity, along with airflow obstruction and remodelling, can lead to the clinical manifestation of symptoms such as dyspnoea, cough, chest tightness and impaired lung function [25].

### Study limitations

This study had a few limitations. First, the sample size was relatively small, and larger sample sizes are essential for robust conclusions. Second, this was a single-centre study, potentially introducing a selection bias. Multicenter studies are warranted to validate these findings in diverse populations. Third, the study design was retrospective, and well-designed prospective studies are necessary to provide more compelling evidence. Despite these limitations, our study provides real-world evidence regarding the AHR in patients with post-COVID. Fourth, the modified Medical Research Council (mMRC) scale is a widely recognised tool for assessing the severity of dyspnoea. In this study, we opted not to use the mMRC scale. Instead, the severity of all reported symptoms, including dyspnoea, was categorised into five levels: none, mild, moderate, severe or very severe, to maintain a uniform approach to symptom evaluation. Despite not using the mMRC scale for dyspnoea, we still provided an assessment of dyspnoea symptoms within the framework of our categorisation.

### Conclusions

This study revealed the prevalence of AHR in a significant majority (43.7%) of post-COVID-19 patients. Post-COVID-19 patients with AHR exhibited distinctive and exacerbated respiratory symptoms, along with a significant decline in PFT parameters after MCT. These findings emphasise the importance of understanding respiratory dynamics, particularly in post-COVID-19 patients with persistent respiratory symptoms.

## Data Availability

The datasets generated during and/or analysed during the current study are available from the corresponding author on reasonable request.
